# Dataset on spatial distribution and location of universities in Nigeria

**DOI:** 10.1016/j.dib.2018.04.093

**Published:** 2018-05-01

**Authors:** G.A. Adeyemi, S.O. Edeki

**Affiliations:** a Department of Civil Engineering, Covenant University, Ota, Nigeria; b Department of Mathematics, Covenant University, Ota, Nigeria

**Keywords:** Educational system, Nigeria universities, Geographic Information System, Data

## Abstract

Access to quality educational system, and the location of educational institutions are of great importance for future prospect of youth in any nation. These in return, have great effects on the economy growth and development of any country. Thus, the dataset contained in this article examines and explains the spatial distribution of universities in the Nigeria system of education. Data from the university commission, Nigeria, as at December 2017 are used. These include all the 40 federal universities, 44 states universities, and 69 private universities making a total of 153 universities in the Nigerian system of education. The data analysis is via the Geographic Information System (GIS) software. The dataset contained in this article will be of immense assistance to the national educational policy makers, parents, and potential students as regards smart and reliable decision making academically.

**Specifications table**

**Table:** 

Subject area	*Education*
More specific subject area	*University settings and locations*
Type of data	*Tables, maps*
How data was acquired	*University commission, GPS coder*
Data format	*Raw and Analysed*
Experimental factors	*Spatial concentration analysis*
Experimental features	*Universities distribution and location*
Data source location	*University commission*, Nigeria
Data accessibility	*Within this article, and*Supplementary files

**Value of the data**

• The data will be of great importance in the Nigerian educational planning since education is a tool of critical value to the well-being of any nation [1], [2].
• The dataset can enhance quality decision making in the educational system.
• The dataset can help to know where new universities are needed.
• The dataset will be of great help in resource management [3].
• Availability of this data as being represented in the maps will enable parents and potential students to easily identify universities within their catchment areas thereby making right decision on their choices of universities [3], [4].
• Publicizing this data will no doubt bring about development mainly in those areas with reasonable number of institutions because investors and business tycoons will be attracted.
• Geographic Information Systems (GIS) enhances qualitative transformational graphic data presentation to enhance quick understanding and accurate decision making in a problem solving environment.

## 1 Data

**Fig. 1:**
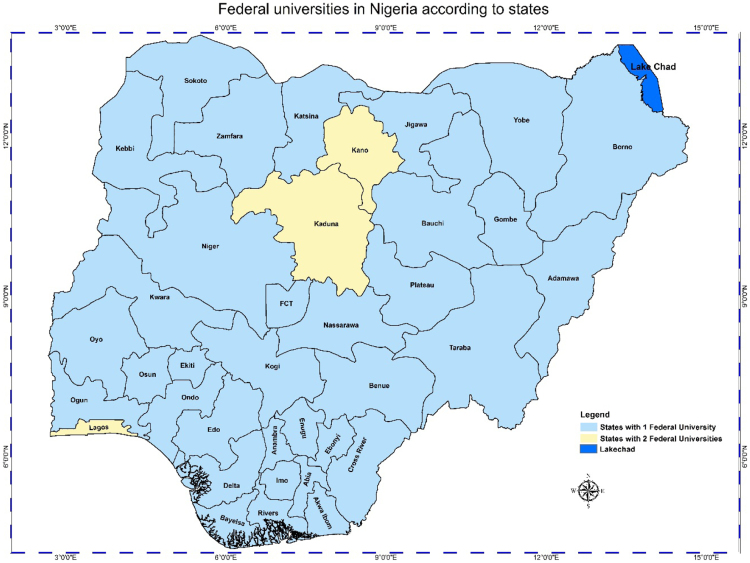
Graphical locations of federal universities in Nigeria.

**Fig. 2:**
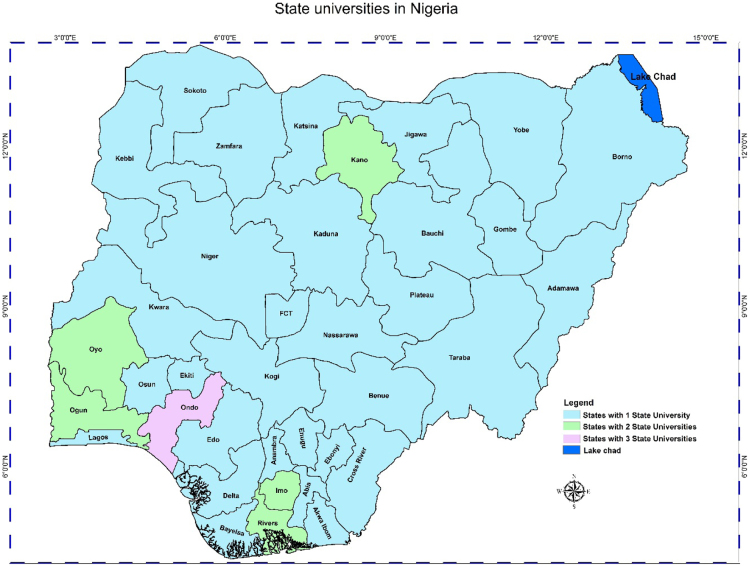
Graphical locations of state universities in Nigeria.

**Fig. 3:**
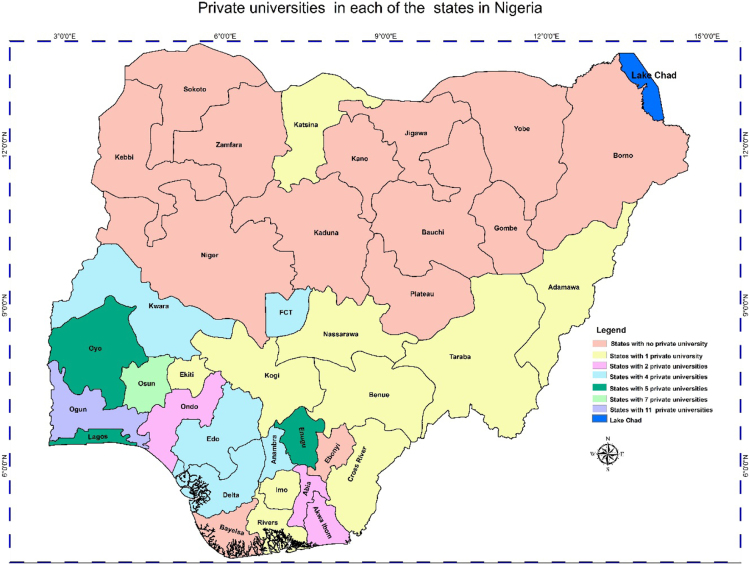
Graphical locations of private universities in Nigeria.

**Fig. 4:**
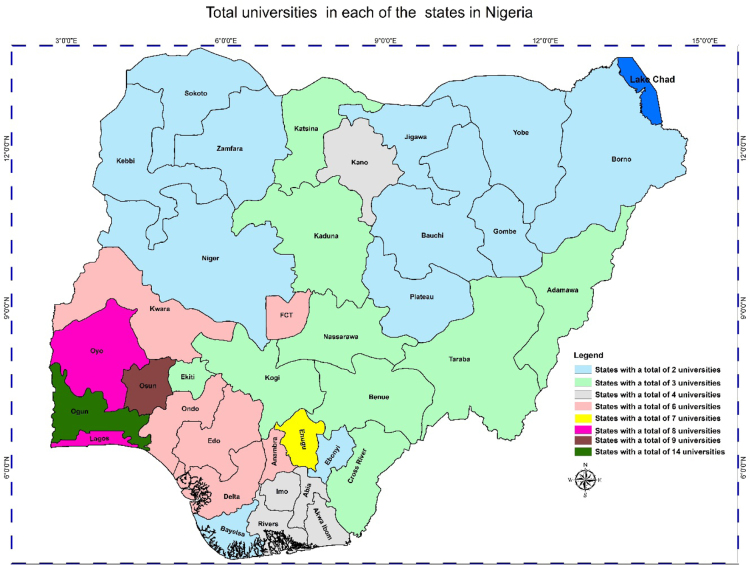
Graphical locations of all universities in Nigeria.

**Fig. 5:**
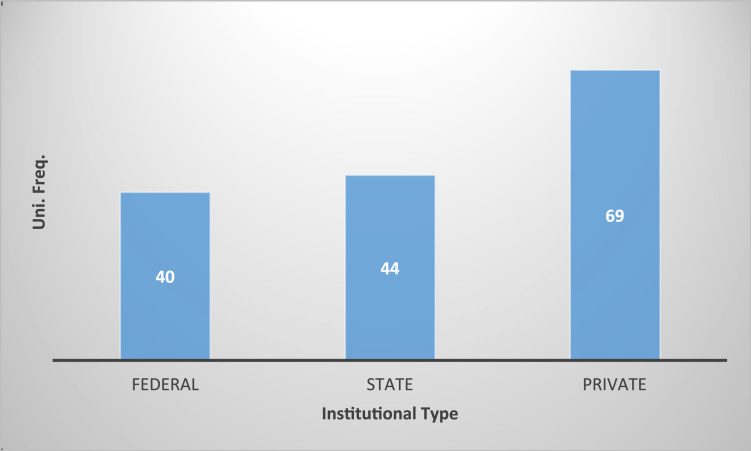
Graphical representation of university-categories in Nigeria.

In any nation, the importance of accessibility to quality educational system, and the location of educational institutions cannot be overlooked. Hence, the need of better descriptive models such as the Geographic Information System (GIS) whose effectiveness in data representation for both qualitative and qualitative decision making in a problem solving environment cannot be over emphasized [5], [6], [7]. This research considers a very distinct way of data representation using a case study of Nigerian Universities. This article contains a total of the 153 universities in the Nigerian system of education as at December 2017. This by nature of institutions are the 40 federal universities, 44 states universities, and 69 private universities. The data analysis is via the Geographic Information System (GIS) software. In Table 1, Table 2, Table 3, we present a concise descriptions of the nature of universities: Federal, state and private (we make reference to Supplementary files). In Fig. 1, Fig. 2, Fig. 3, Fig. 4, the maps obtained via the GIS software are presented. Fig. 5 contains the graphical representation of University-categories in Nigeria.

## 2 Experimental design, materials and methods

### 2.1 Data analysis

See Table 1, Table 2, Table 3 and Fig. 1, Fig. 2, Fig. 3, Fig. 4, Fig. 5.

**Table 1:** Federal universities in the Nigeria setting.

Federal universities in number	Concerned States in number	**Frequency**
1	34	34
2	3	6
**Cumulative**		**40**

**Table 2:** State universities in the Nigeria setting.

State universities in number	Concerned States in number	**Frequency**
1	31	31
2	05	10
3	01	03
**Cumulative**		**44**

**Table 3:** Private universities in the Nigeria setting.

Private universities in number	Concerned States in number	**Frequency**
1	10	10
2	3	06
4	5	20
5	3	15
7	1	07
11	1	11
**Cumulative**		**69**

### 2.2 A note on the analysis

From Table 1, there are 40 federal universities while 2 states have 3 federal universities each and 34 have 1 federal university each. Note: the Federal Capital Territory (FCT) is included. In Table 2, we have 31 states with 1 state university each, 5 states with 2 state universities each and 1 state with 3 state universities. In Table 3 we have 10, 3, 5, 3, 1, and 1 state(s) with 1, 2, 4, 5, 7, and 11 private universities respectively. It is obvious from the information (Table 3 and Fig. 3) that most states do not have private universities.

### 2.3 Effects on national growth: unemployment and underemployment

Low level of education due to reasons such as personal interest, lack of awareness in terms of educational importance, poor educational background, and so on has in no doubt affected the employment level of the country leading to adverse effects in area of national growth and development. According to Labor Force Statistics Vol 1: *unemployment and underemployment report* (NBS 2017) [8], states like Kastina, Jigawa, Gombe and Yobe witnessed highest underemployment rates during the concerned period of exercise 2017. The recorded percentages (%) for these states are 46.19%, 43.01%, 39.06%, and 38.38% respectively (Fig. 3). On the other hand, Rivers state, Akwa-Ibom state, Bayelsa state, and Imo state recorded highest level of unemployment with the respective percentages as follows: 41. 82%, 36.58%, 30.36%, and 29.47% (Fig. 4). This is traceable to the effect of migration caused by the less educational privilege states. The effect of volatility on the economic growth and development of any nation cannot be ignored: this include macroeconomic volatility in relation to finance and education [9], [10], [11], [12], [13], [14], [15].
